# Changing levels of local crime and mental health: a natural experiment using self-reported and service use data in Scotland

**DOI:** 10.1136/jech-2020-213837

**Published:** 2020-10-01

**Authors:** Gergő Baranyi, Mark Cherrie, Sarah E Curtis, Chris Dibben, Jamie Pearce

**Affiliations:** 1 Center for Research on Environment, Society and Health, School of GeoSciences, University of Edinburgh, Edinburgh, UK; 2 Geography Department, Durham University, Durham, UK

**Keywords:** MENTAL HEALTH, CRIME, NATURAL EXPERIMENT, ANTIPSYCHOTICS, ANTIDEPRESSANTS, NEIGHBOURHOOD

## Abstract

**Background:**

This study contributes robust evidence on the association between mental health and local crime rates by showing how changing exposure to small area-level crime relates to self-reported and administrative data on mental health.

**Methods:**

The study sample comprised 112 251 adults aged 16–60 years, drawn from the Scottish Longitudinal Study, a 5.3% representative sample of Scottish population followed across censuses. Outcomes were individual mental health indicators: self-reported mental illness from the 2011 Census and linked administrative data on antidepressants and antipsychotics prescribed through primary care providers in the National Health Service in 2010/2012. Crime rates at data zone level (500–1000 persons) were matched to the participants’ main place of residence, as defined by general practitioner patient registration duration during 2004/2006, 2007/2009 and 2010/12. Average neighbourhood crime exposure and change in area crime were computed. Covariate-adjusted logistic regressions were conducted, stratified by moving status.

**Results:**

In addition to average crime exposure during follow-up, recent increases in crime (2007/2009–2010/2012) were associated with a higher risk of self-reported mental illness, among ‘stayers’ aged 16–30 years (OR=1.11; 95% CI 1.00 to 1.22), and among ‘movers’ aged 31–45 years (OR=1.07; 95% CI 1.01 to 1.13). Prescribed medications reinforced these findings; worsening crime rates were linked with antidepressant prescriptions among young stayers (OR=1.09; 95% CI 1.04 to 1.14) and with antipsychotic prescriptions among younger middle-aged movers (OR=1.11; 95% CI 1.01 to 1.23).

**Conclusion:**

Changing neighbourhood crime exposure is related to individual mental health, but associations differ by psychiatric conditions, age and moving status. Crime reduction and prevention, especially in communities with rising crime rates, may benefit public mental health.

## INTRODUCTION

Mental disorders are major contributors to the global disease burden and present the leading cause of disability among young adults.^1^ In high-income countries, the burden is even larger: one in six adults in the United Kingdom is affected by common mental disorders at any given time,^2^ causing direct and indirect costs that equate to over 4% of the national gross domestic product.^3^ Over and above individual biopsychosocial determinants, the physical and social environment where people live influences mental health.^4–6^

Residential areas with high levels of deprivation and social disorganisation tend to have more crime and violence,^7 8^ impacting mental health due to heightened risks of personal victimisation and witnessing crime,^9^ and through an ecological pathway by inducing stress and fear of crime.^10^ While the notion that neighbourhood-level crime is associated with self-reported symptoms and mental health service use has been confirmed in ecological,^11^ cross-sectional^,^^12 13^ and longitudinal^14–16^ studies, investigations examining spatial and temporal variation in exposure to crime are lacking. Crime is not randomly distributed; incidents in a small number of micro-geographic areas account for a large proportion of total crime,^8 12^ which can be key to explaining the relationship with mental health.^12^

Individual exposure to residential characteristics may change as the surrounding area alters in response to political and other contextual influences (eg, revitalisation, gentrification, postindustrial decline),^17^ or through residential mobility by people moving to different areas. Increasing neighbourhood deprivation has been linked to distress among residents staying in the same area.^18^ Also, moving from high-poverty to low-poverty areas might be beneficial for mental health, as demonstrated in experimental^19^ and observational studies.^20^ While there is some evidence that rising crime can be detrimental to mental health,^14 15^ studies often use crime aggregated into large geographic units, which may lack the specificity to capture spatio-temporal variability in crime.^12^ Finally, not only may neighbourhood crime cause mental disorders, but people with disadvantaged background or with pre-existing health conditions might be more likely to move into higher crime areas.^21^

Since the 1990s, the national-level reported crime rate has dropped in Scotland; however, the reductions have not been uniform between communities,^8^ providing an opportunity to use the spatio-temporal variation in crime as a natural experiment.^14^ To address this research gap, we investigated how individual self-reported mental illness and prescribed psychotropic medications related to increasing neighbourhood crime levels, taking into account residential mobility. Moreover, we aimed to identify demographic groups whose mental health seemed most vulnerable to crime effects, where prevention and service development might be particularly beneficial.

## METHODS

### Sample

**Figure 1:**
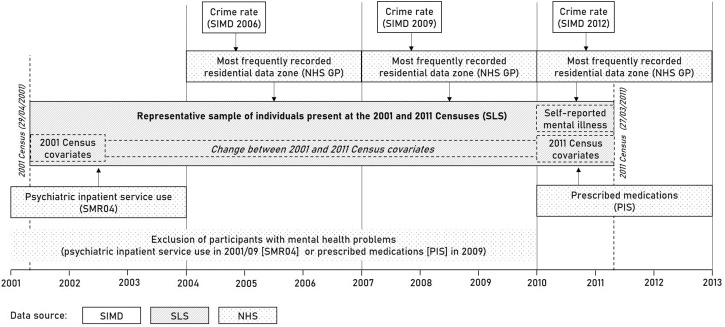
Operationalising crime, covariate and mental health variables using longitudinal data linkage in Scotland (2001-2013). Crime rates reported in the 2006/2009/2012 Scottish Index of Multiple Deprivation (SIMD) were linked to the Scottish Longitudinal Study (SLS) by using the residential data zone where SLS members were registered for the longest time during 2004/06, 2007/09 and 2010/12. Residential location was derived from records of the National Health Service (NHS) general practitioner (GP) registration database. Mental health service use within the NHS system was extracted from the Scottish National Prescription Information System (PIS) and from the Scottish Morbidity Records (SMR04), and information was linked to SLS participants using unique personal identifiers. For sensitivity analysis, participants with any records of mental health service use between 2001 and 2009 (SMR04 & PIS) were excluded from the sample.

Data were drawn from the Scottish Longitudinal Study (SLS), a 5.3% nationally representative sample of the Scottish population. The SLS includes individuals selected on the basis of 20 semirandom birthdates and present in any of the 1991, 2001 and 2011 Censuses.^22^ For this study, a subsample of >126 000 was extracted, including individuals present at both the 2001 and 2011 Censuses and aged between 16 and 60 years in 2001. We applied age restrictions because psychopharmacological treatment among older adults may be less likely to be initiated by a mental disorder.^16 23^ Individuals were excluded if living in communal establishments (eg, nursing homes) in 2001 or 2011 (1.0%) or having missing values for the covariates (10.5%). Area-level indicators of crime were linked to SLS participants using residential localities and dates of their registration with a general practitioner (GP). Healthcare administrative data were linked to participants based on unique personal identifiers (figure 1).

### Measures

#### Mental health indicators

Mental health was measured using information on self-reported mental illness and prescribed medications. In 2011, all census respondents were asked whether they had ‘*…*conditions which have lasted, or are expected to last, at least 12 months?’, with various response categories including ‘mental health condition’, taken here to indicate self-reported mental illness.

NHS administrative data on prescriptions for antidepressants (British National Formulary 4.3) and antipsychotics (British National Formulary 4.2) were derived from the Scottish National Prescription Information System, which covers all NHS Scotland prescriptions, prescribed, dispensed and reimbursed in the community setting.^24^ Antidepressants are mainly used to treat moderate-to-severe depression and in some cases anxiety disorders. At low dosage (≤30 mg per day), amitriptyline and nortriptyline are often prescribed for neurological conditions, so these low-dose prescriptions were excluded from our study.^25^ Antipsychotics are principally used to treat psychotic and related disorders; however, severe anxiety can be also treated with them in the short term.^25^ Individuals with at least six prescriptions for antidepressants or antipsychotics in 2010/2012 were defined as cases.^16^

Self-reported mental health and prescribed medications are not available prior to 2009. To control for mental illness at baseline, psychiatric inpatient service use in 2001/2003 for substance use, psychotic, mood and neurotic disorders (ICD-10 codes: F10-F48) from the Mental Health Inpatient & Day Case dataset (Scottish Morbidity Records, SMR04) of NHS Scotland were linked to SLS.^26^

#### Neighbourhood crime

**Figure 2:**
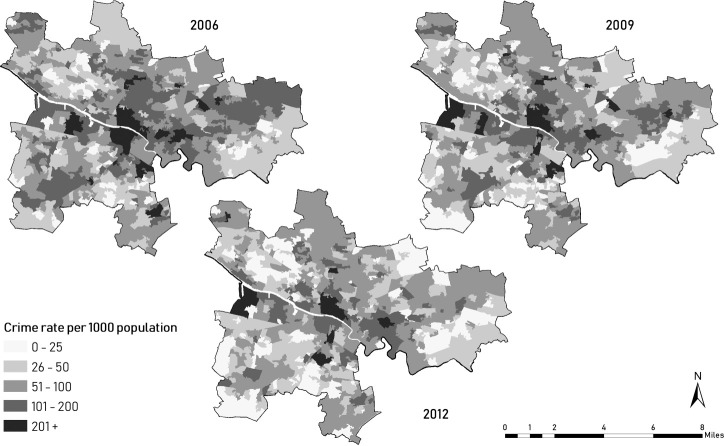
Crime rates per 1000 population in Glasgow City, Scotland, as reported in the 2006, 2009 and 2012 Scottish Index of Multiple Deprivation.

The Scottish Index of Multiple Deprivation (SIMD) includes a domain on local crime and is available for 6505 Scottish data zones, each comprising approximately 500–1000 residents. The crime domain aggregates police recorded and geo-referenced crimes and offences (eg, assault, crimes of violence, domestic housebreaking, drug offences and vandalism) throughout the preceding financial year.^27^ The Scottish Government applies disclosure control in low-crime areas by suppressing exact crime counts. To approximate missing values, we first ordered data zones by their non-suppressed crime ranks, assigned 0 crime into the lowest ranked unit and used linear interpolation to estimate suppressed numbers. Finally, crime rates per 1000 persons were computed based on population estimates. SIMD 2006 (first release), 2009 and 2012 provided longitudinal information on crime with consistent data zone boundaries (see changes in crime levels for Glasgow City in figure 2).

To link neighbourhood crime to SLS members, we used data on places of residence from the NHS GP registration database, holding records on patient registrations with GPs from 2000 onwards.^28^ The SLS team derived for each participant the residential history comprising all residential data zones and dates of the changes recorded during the study. We assigned each SIMD crime release to a 3-year time interval (2004/2006 for SIMD 2006, 2007/2009 for SIMD 2009 and 2010/2012 for SIMD 2012), extracted for each participant the main residential data zone where the participants were registered for the longest time within these three intervals and linked the participants to their respective crime rates. Finally, we stratified the sample into subsets, comprising individuals for whom the main residential data zone did not change during the study (stayers), changed between 2007/2009 and 2010/2012 (recent movers) and changed between 2004/2006 and 2007/2009 (past movers).

#### Covariates

Covariates were derived from the 2001 and 2011 Censuses. Age and sex, extracted from both censuses, were reviewed for consistency before inclusion. Baseline variables were derived from the 2001 Census and classified as follows: ethnicity (white, non-white); highest educational attainment (no qualification, levels 1–4); social class based on occupation (I/II, IIIN, IIIM, IV, V); employment status (employed, unemployed, retired, out of labour force, student); marital status (married, single, separated, divorced, widowed); living status (alone, with others); and limiting long-term illness (yes, no). For time-variant covariates, we computed binary change indicators between censuses (change, no change): gained higher level of education; separated, divorced or widowed; started to live alone; and became unemployed or left labour force. There is no consistent social class measure in 2001 and 2011 due to differences in census questions/codings; therefore, we included also the 2011 social grade variable (AB, C1, C2, D, E) in the models. A detailed description of the covariates is in Supplementary Table 1.

10.1136/jech-2020-213837.supp1 Supplementary data

**Table 1:** Individual characteristics among the sample of 112 251 Scottish adults (%)

Variable		Value	%
Moving status		Stayer (2004/2006–2010/2012)	72
		Past mover (2004/2006–2007/2009)	14
		Recent mover (2007/2009–2010/2012)	14
2001 covariates	Sex	Male	47
		Female	53
	Age cohorts	16–30	27
		31–45	40
		46–60	33
	Ethnicity	White	99
		Non-white	1
	Highest educational attainment	No qualifications	29
		Level 1	28
		Level 2	16
		Level 3	8
		Level 4	20
	Social class based on occupation	I/II—Professional, managerial and technical occupations	33
		IIIN—Skilled non-manual occupations	24
		IIIM—Skilled manual occupations	19
		IV—Partly skilled occupations	15
		V—Unskilled occupations	6
		Other	4
	Employment status	Employed	73
		Unemployed	4
		Student	6
		Retired	2
		Out of labour force	15
	Marital status	Single	33
		Married	55
		Separated	4
		Divorced	7
		Widowed	1
	Living status	Alone	11
		With others	89
	Long-term illness	No	87
		Yes	13
	Psychiatric inpatient service use in 2001/2003	No	99
		Yes	1
2011 covariate	Social grade	AB—Higher or intermediate managerial, administrative or professional grade	21
		C1—Supervisory, clerical and junior managerial, administrative and professional grade	31
		C2—Skilled manual workers	25
		D—Semiskilled and unskilled manual workers	22
		E—State pensioners, casual and lowest grade workers, unemployed with state benefits only	3
2001–2011 change indicators	Education	No change	77
		Gained education between 2001 and 2011	23
	Employment	No change	95
		Became unemployed or left labour force between 2001 and 2011	5
	Marital status	No change	93
		Separated, divorced or widowed between 2001 and 2011	7
	Living status	No change	91
		Started to live alone between 2001 and 2011	9

Source: Scottish Longitudinal Study.

Note: Percentages are presented in whole numbers to avoid risk of disclosure; they may not sum to 100% because of rounding errors.

### Statistical analysis

While repeatedly measured predictors were available, outcomes were only assessed at the end of the study. To preserve the longitudinal nature of the data, for each participant, we calculated summary measures^29^ of neighbourhood crime by decomposing average exposure during follow-up and change in exposure to crime. For average crime exposure ($\bar{x}$), first the arithmetic mean of the crime rates was calculated and then log-transformed in order to minimise the effect of extreme outliers and right-skewed distribution (equation 1). Change in crime exposure variables ($x_{\Delta }$) was computed as the standardised difference between the person’s average of crime exposure and the crime rates of places they lived in 2004/2006 or 2010/2012, with positive values expressing increasing rates and negative values expressing decreasing rates (equations 2, 3). While average crime rates were related to long-term differences between individuals, change in crime indicated within-individual variation in exposure.

1 $$\bar{x}=\log10\left(\frac{x_{2004/06}+x_{2007/09}+x_{2010/12}}{3}\right)$$

2 $$x_{\Delta 2004/06}=sdr\left(\frac{x_{2004/06}+x_{2007/09}+x_{2010/12}}{3}-x_{2004/06}\right)$$

3 $$x_{\Delta 2010/12}=sdr\left(x_{2010/12}-\frac{x_{2004/06}+x_{2007/09}+x_{2010/12}}{3}\right)$$

We fitted logistic regression models with clustered robust estimations, allowing SEs varying between the 32 Scottish local authorities^16^ as recorded at the time of outcome measurement. All models included average and change variables. In the first set of models, we controlled for sex, age and age-squared. The second model additionally adjusted for all 2001 covariates (ethnicity, education, social class, employment, marital status, living status, long-term illness) and for psychiatric inpatient service use in 2001/2003. Finally, in the fully adjusted model, we additionally controlled for changing individual circumstances between 2001 and 2011 (gained higher level of education; separated, divorced or widowed; started to live alone; became unemployed or left labour force) and for social grade in 2011. Models were run separately for those identified as residential ‘movers’ and ‘stayers’. For past movers, we included the 2004/2006 ($x_{\Delta 2004/06})$ and for stayers and recent movers the 2010/2012 change variable ($x_{\Delta 2010/12}$). As the effect of crime might differ by age,^16^ models were presented separately in young adulthood (aged 16–30 years in 2001), younger middle adulthood (aged 31–45 years) and older middle adulthood (aged 46–60 years).

The following sensitivity analyses were carried out. (1) Using the same method as for crime, we extracted data zone income deprivation from the 2006/2009/2012 SIMDs, calculated standardised average and standardised change of deprivation and imputed them in the final models, in order to test whether crime change had a robust effect over and above income deprivation. (2) Instead of extracting the main residential data zone in each interval, we restricted the stayer subsample to those who lived at the same location during all 108 months of the study. (3) In order to strengthen the causal perspective, we excluded from the sample all individuals who were likely to have long-standing mental health conditions prior to outcome measurement, indicated by those who had any psychiatric admission in 2001/2009 and any psychotropic prescription in 2009 (medication data are not available prior to 2009)^24^ (figure 1). For this analysis, Poisson regression with clustered robust SEs estimated the incidence rate ratio (IRR) of crime on mental ill health.

## RESULTS

Out of 112 251 Scottish adults, 72% were classified as ‘stayers’, 14% as ‘past movers’ and 14% as ‘recent movers’ (table 1). At the end of the study, 5.0% of the sample reported having a long-term mental illness, 14.4% had been prescribed at least six rounds of antidepressants and 1.2% had at least six rounds of antipsychotic prescriptions. The prevalence of mental health outcomes differed across moving status and age cohorts, with higher rates among middle-aged adults and recent movers, especially for antipsychotics (Supplementary Table 2). For the total sample, the average neighbourhood crime rate was 44.2 per 1000 persons (SD=47.1); the crime rate dropped by 5.7 (SD=30.3) between 2004/2006 and 2007/2009 and by 6.9 per 1000 persons (SD=25.5) between 2007/2009 and 2010/2012. Young adults and recent movers were exposed to higher neighbourhood crime on average, but they also experienced a larger drop in exposure (Supplementary Table 3).

**Table 2:** Associations between average crime exposure, change in crime exposure and mental health outcomes

	Total sample (n=112 251)		Stayers (n=80 958)		Past movers† (n=15 940)		Recent movers‡ (n=15 353)	
	Average crime exposure ($\bar{x}$)	Change in crime exposure ($x_{\Delta 2010/12}$)	Average crime exposure ($\bar{x}$)	Change in crime exposure ($x_{\Delta 2010/12}$)	Average crime exposure ($\bar{x}$)	Change in crime exposure ($x_{\Delta 2004/06}$)	Average crime exposure ($\bar{x}$)	Change in crime exposure ($x_{\Delta 2010/12}$)
**Self-reported mental health**								
Model 1§	**3.44 (2.87–4.13)**	**1.05 (1.04–1.07)**	**3.12 (2.50–3.90)**	**1.09 (1.00–1.18)**	**3.71 (2.96–4.65)**	1.02 (1.00–1.04)	**4.55 (3.36–6.16)**	**1.06 (1.03–1.08)**
Model 2¶	**1.79 (1.60–2.00)**	**1.04 (1.02–1.06)**	**1.57 (1.38–1.77)**	1.03 (0.95–1.11)	**2.00 (1.64–2.45)**	1.03 (1.00–1.06)	**2.28 (1.71–3.02)**	**1.05 (1.02–1.08)**
Model 3**	**1.51 (1.35–1.68)**	**1.04 (1.02–1.06)**	**1.40 (1.24–1.57)**	1.02 (0.94–1.11)	**1.56 (1.27–1.91)**	1.02 (0.99–1.05)	**1.77 (1.33–2.36)**	**1.04 (1.01–1.07)**
**Antidepressant prescriptions**								
Model 1§	**1.98 (1.85–2.12)**	**1.03 (1.01–1.05)**	**1.89 (1.76–2.03)**	**1.05 (1.02–1.08)**	**1.90 (1.65–2.20)**	1.00 (0.99–1.02)	**2.44 (2.09–2.86)**	**1.03 (1.01–1.06)**
Model 2¶	**1.37 (1.30–1.45)**	1.02 (1.00–1.04)	**1.32 (1.24–1.40)**	1.01 (0.98–1.04)	**1.29 (1.11–1.50)**	1.00 (0.98–1.03)	**1.55 (1.32–1.83)**	1.02 (0.99–1.05)
Model 3**	**1.27 (1.20–1.34)**	1.01 (0.99–1.03)	**1.25 (1.17–1.34)**	1.01 (0.98–1.04)	1.16 (0.99–1.36)	1.00 (0.98–1.03)	**1.32 (1.12–1.56)**	1.01 (0.98–1.04)
**Antipsychotic prescriptions**								
Model 1§	**3.30 (2.76–3.96)**	**1.06 (1.03–1.10)**	**3.16 (2.48–4.01)**	**1.16 (1.04–1.29)**	**2.98 (1.95–4.56)**	1.01 (0.98–1.05)	**4.70 (3.11–7.10)**	**1.06 (1.03–1.09)**
Model 2¶	**1.42 (1.22–1.67)**	**1.07 (1.02–1.12)**	**1.34 (1.08–1.67)**	1.10 (0.98–1.24)	1.14 (0.69–1.91)	1.04 (0.97–1.11)	**2.15 (1.34–3.44)**	**1.06 (1.01–1.11)**
Model 3**	**1.25 (1.06–1.47)**	**1.06 (1.01–1.12)**	**1.24 (1.00–1.54)**	1.10 (0.98–1.24)	0.94 (0.58–1.52)	1.03 (0.97–1.10)	**1.65 (1.02–2.69)**	1.05 (0.99–1.12)

Source: Scottish Longitudinal Study.

Note: Bold text indicates significant associations (*p*<0.05). Models were fitted with logistic regression applying cluster robust estimation at local authority level; estimates are expressed in OR with 95% CI. Average crime exposure is log10-transformed, change in crime exposure is standardised. Models included average and change variables at the same time.

†Main residential location changed between 2004/2006 and 2007/2009.

‡Main residential location changed between 2007/2009 and 2010/2012.

§Model 1: Controlled for sex, age and age-squared.

¶Model 2: Model 1+2001 baseline covariates (ethnicity, education, social class, employment, marital status, living status, long-term illness) and psychiatric inpatient service use in 2001/2003.

**Model 3: Model 2+2001–2011 change indicators (gained higher level of education; separated, divorced or widowed; started to live alone; became unemployed or left labour force) and social grade in 2011.

**Table 3:** Age cohort-specific associations between average crime exposure, change in crime exposure and mental health outcomes

	Total sample (n=112 251)		Stayers (n=80 958)		Past movers† (n=15 940)		Recent movers‡ (n=15 353)	
	Average crime exposure ($\bar{x}$)	Change in crime exposure ($x_{\Delta 2010/12}$)	Average crime exposure ($\bar{x}$)	Change in crime exposure ($x_{\Delta 2010/12}$)	Average crime exposure ($\bar{x}$)	Change in crime exposure ($x_{\Delta 2004/06}$)	Average crime exposure ($\bar{x}$)	Change in crime exposure ($x_{\Delta 2010/12}$)
**Self-reported mental-health**								
16–30 years old	**1.84 (1.54–2.21)**	**1.03 (1.01–1.05)**	**1.92 (1.54–2.41)**	**1.11 (1.00–1.22)**	1.34 (0.99–1.83)	1.02 (0.97–1.08)	**2.18 (1.49–3.20)**	1.02 (0.99–1.05)
31–45 years old	**1.41 (1.20–1.65)**	**1.05 (1.01–1.10)**	**1.26 (1.03–1.54)**	1.01 (0.91–1.12)	**1.89 (1.20–2.98)**	1.04 (0.99–1.09)	**1.60 (1.09–2.34)**	**1.07 (1.01–1.13)**
46–60 years old	**1.32 (1.10–1.58)**	1.01 (0.97–1.05)	**1.27 (1.06–1.52)**	0.97 (0.88–1.07)	1.49 (0.87–2.54)	1.03 (0.98–1.09)	1.18 (0.64–2.16)	1.04 (0.96–1.12)
**Antidepressant prescriptions**								
16–30 years old	**1.30 (1.16–1.45)**	1.01 (0.99–1.04)	**1.41 (1.21–1.64)**	**1.09 (1.04–1.14)**	1.06 (0.83–1.35)	1.01 (0.97–1.04)	1.29 (0.99–1.67)	1.01 (0.97–1.04)
31–45 years old	**1.24 (1.14–1.34)**	1.01 (0.98–1.05)	**1.18 (1.05–1.33)**	1.01 (0.94–1.07)	1.22 (0.97–1.53)	1.01 (0.98–1.05)	**1.54 (1.16–2.05)**	1.01 (0.98–1.05)
46–60 years old	**1.24 (1.13–1.36)**	1.00 (0.97–1.04)	**1.24 (1.13–1.36)**	0.98 (0.94–1.02)	1.20 (0.79–1.83)	0.96 (0.91–1.02)	0.93 (0.63–1.36)	1.03 (0.97–1.11)
**Antipsychotic prescriptions**								
16–30 years old	1.19 (0.93–1.52)	1.01 (0.92–1.10)	1.26 (0.80–1.96)	1.23 (0.88–1.73)	0.98 (0.50–1.95)	1.04 (0.97–1.12)	1.32 (0.71–2.44)	0.96 (0.89–1.03)
31–45 years old	1.30 (0.95–1.77)	1.10 (1.00–1.20)	1.14 (0.79–1.63)	1.04 (0.89–1.22)	0.97 (0.45–2.08)	1.07 (0.99–1.16)	**2.44 (1.20–4.97)**	**1.11 (1.01–1.23)**
46–60 years old	1.22 (0.90–1.66)	1.08 (0.97–1.20)	1.31 (0.93–1.84)	0.12 (0.93–1.34)	0.71 (0.18–2.87)	0.98 (0.86–1.12)	0.73 (0.22–2.38)	1.11 (1.00–1.23)

Source: Scottish Longitudinal Study.

Note: Age cohorts relate to age in 2001. Bold text indicates significant associations (*p*<0.05). Average crime exposure is log10-transformed, change in crime exposure is standardised. Models were fitted with logistic regression applying cluster robust estimation at local authority level; estimates are expressed in OR with 95% CI. Models included average and change variables at the same time and were adjusted for sex, age (and age-squared in the non-stratified total sample), 2001 baseline covariates (ethnicity, education, social class, employment, marital status, living status, long-term illness), psychiatric inpatient service use in 2001/2003, 2001–2011 change indicators (gained higher level of education; separated, divorced or widowed; started to live alone; became unemployed or left labour force) and social grade in 2011.

†Main residential location changed between 2004/2006 and 2007/2009.

‡Main residential location changed between 2007/2009 and 2010/2012.

### Self-reported mental illness

In the fully adjusted models, in addition to a strong association with higher average crime exposure (OR=1.51; 95% CI 1.35 to 1.68), 1 SD increase in crime was associated with 4% higher odds of reporting mental illness (95% CI 1.02 to 1.06). In the models stratified by moving status, crime increase remained significant only among recent movers (OR=1.04; 95% CI 1.01 to 1.07) (table 2). After stratifying by age cohorts, the association with average crime exposure was stronger among younger individuals (OR=1.84; 95% CI 1.54 to 2.21). Moreover, 1 SD increase in crime exposure elevated the odds of self-reported mental illness by 11% (95% CI 1.00 to 1.22) among young stayers (due to change in local crime rates) and by 7% (95% CI 1.01 to 1.13) among recently moved younger middle-aged adults (table 3).

### Prescribed medications

Higher average crime exposure increased the risk of having been prescribed at least six rounds of antidepressants (OR=1.27; 95% CI 1.20 to 1.34) or antipsychotics (OR=1.25; 95% CI 1.06 to 1.47), with associations being stronger among recent movers. Change in crime exposure, however, only remained significant for antipsychotics in the fully adjusted models (OR=1.06; 95% CI 1.01 to 1.12) (table 2). When exploring association by age cohorts, models of prescribed medications reinforced the findings for self-reported mental illness (table 3): 1 SD increase in crime exposure among young stayers increased the odds of antidepressant prescriptions by 9% (95% CI 1.04 to 1.14); among younger middle-aged movers, it increased the odds of antipsychotic prescriptions by 11% (95% CI 1.01 to 1.23).

### Sensitivity analyses

After further adjustment for income deprivation, associations with average crime exposure only remained significant among older middle-aged stayers for antipsychotics and among young stayers for self-reported mental illness. Associations with change in exposure to crime were substantially attenuated for self-reported mental illness; however, they did not materially change for antidepressant and antipsychotic prescriptions (Supplementary Table 4). Findings on individuals staying all 108 months of the study at the same location reinforced that young adults were more vulnerable to increasing crime rates, with elevated risk of self-reported mental illness and antidepressant prescriptions (Supplementary Table 5). Finally, after excluding participants with mental health service use between 2001 and 2009, the prevalence of self-reported mental illness, antidepressant and antipsychotic prescriptions during 2010/2012 dropped with 80%, 74% and 92%, respectively; drops in cases were particularly pronounced among middle-aged adults. The findings in this reduced sample confirmed previous associations for antidepressant medications among young stayers (IRR=1.12; 95% CI 1.04 to 1.21). For antipsychotics, increasing crime exposure among young stayers significantly predicted prescriptions (IRR=1.59; 95% CI 1.07 to 2.37), while the substantial drop in cases precluded analyses among movers (Supplementary Table 6).

## DISCUSSION

This study provides a longitudinal perspective on the association between long-term average neighbourhood crime exposure and recent changes in crime and mental health in Scotland, using a natural experimental framework. Associations between average crime exposure and self-reported mental illness were twice as strong as for prescriptions and were mainly driven by relationships for the youngest age group. Recent increases in crime rates were related to mental health in two population subgroups: for self-reported mental illness and antidepressants among young adults staying in the same neighbourhood and for self-reported mental illness and antipsychotics among recently moved younger middle-aged adults. Sensitivity analyses reinforced the findings on antidepressants, but they challenged the causal perspective for antipsychotics.

This study extends the literature on the longitudinal relationships between neighbourhood crime and mental health,^14–16^ by estimating the link for self-reported versus service use outcomes, and in different age cohorts. Stronger association between average crime exposure and self-reported mental illness, in comparison to prescribed medications, may reflect how the former variable was measured. Self-reported mental illness might capture more serious and long-standing problems and thus had a lower prevalence than antidepressant prescriptions. It is also plausible that using psychotropic medications underestimated the association with crime by not fully capturing affected individuals from lower socioeconomic groups^2^ and including prescriptions not related to mental illness.^25^ Moreover, while the gap between mental health need and treatment is disproportionately large among young adults with low use of medications,^2^ they are more often victims of crime.^30^ This may explain the stronger links between crime exposure and self-reported mental illness in this cohort.

In comparison to average neighbourhood crime exposure, changes in individual exposure are less likely to be affected by residual confounding and may strengthen the causal evidence between exposure and outcome. Associations with increasing crime were evident in younger age, confirmed by both self-reported and medication data. Because of higher frequency of victimisation,^30^ young adults remaining in the same neighbourhood may be more vulnerable to increasing crime in their locality, linked to mental health conditions treated with antidepressants. Sensitivity analyses confirmed this link by supporting that the causation hypothesis^31^ may provide a suitable explanation for the neighbourhood-level crime and depression association.

Associations with changing crime exposure for antipsychotic prescriptions were more complex. After excluding individuals with pre-existing psychiatric conditions, the previously robust association among movers could not be estimated because of the large drop in cases. It is plausible that findings among middle-aged individuals (for whom the highest incidence rate of first-episode psychosis in young adulthood has already passed)^32^ reflect selective migration into higher crime areas related to pre-existing severe mental disorders.^21^ Moreover, the increased risk of antipsychotic medication among young adults staying in the same area may require further exploration, as there is evidence suggesting that growing up in high-crime neighbourhoods may increase the risk of presenting psychotic symptoms through increased social stress and crime victimisation.^33 34^

This longitudinal data linkage study benefitted from a large and representative sample, covering the entire country with very low attrition rates.^22^ The NHS is (effectively) universally used in Scotland and psychotropic prescriptions were routinely collected with an exceptional completeness (95% of reimbursed prescriptions within NHS Scotland are captured with unique personal identifiers).^24^ However, several limitations have to be considered. First, while the NHS GP registration database contains residential localities with high accuracy, the reliability of the data might differ across age and clinical groups. Second, in Scotland, only 40% of crimes are reported to the police,^30^ which may introduce bias to our findings. Third, outcomes were not available prior to 2009, precluding more robust statistical analyses (eg, fixed-effects models).^14^ Finally, self-reported mental illness and prescription data cannot be directly linked to psychiatric conditions; further studies with specific diagnoses are required to break down the neighbourhood crime–mental health relationship.

In conclusion, neighbourhood-level crime is a significant determinant of mental health and requires system-based actions. Crime reduction through neighbourhood interventions^35^ and spatially targeted policing^36^ may be beneficial for population mental health, particularly for young adults. Delivering mental health promotion for young people in high-crime areas, for example, school-based preventions^37^ and indicative prevention for high-risk individuals,^38^ as well as allocating services (eg, early psychosis programmes)^39^ to the vicinity of high-crime areas, may improve mental health and reduce the associated societal and economic burden.

**What is already known on this subject**

- Living in a high-crime area is related to mental health problems, but there are very few longitudinal studies using repeated measurements of small area-level crime rates.
- There is little research on whether this relationship differs by moving status, psychiatric conditions or age groups.

**What this study adds**

- For young adults staying in the same neighbourhood, increasing crime rates in their residential area were positively associated with self-reported mental illness and antidepressant prescriptions.
- Among recently residentially mobile younger middle-aged adults, moving to higher crime areas was associated with greater risk of self-reported mental illness and antipsychotic prescriptions; these findings were likely explained to some extent by health selective migration.
- Neighbourhood interventions, targeted policing and delivering mental health preventions and services in high-crime areas may help to reduce inequalities in mental health.
