# Supplementary material for: Changing levels of local crime and mental health: a natural experiment using self-reported and service use data in Scotland
Source: J Epidemiol Community Health. 2020 Oct 1;74(10):806–14. doi: 10.1136/jech-2020-213837 (PMC7577093; doi:10.1136/jech-2020-213837)
Supplement: Supplementary data [file jech-2020-213837s001.pdf]

Changing levels of local crime and mental health: A natural experiment utilising self-reported and service use data in Scotland

Gergő Baranyi, Mark Cherrie, Sarah Curtis, Chris Dibben & Jamie Pearce

Supplementary Materials

Supplementary Table 1: Description of the covariates.

Supplementary Table 2: Sample size and percentage of individuals with mental health problems by age and moving status.

Supplementary Table 3: Average crime exposure and change in crime by age and moving status (crime per 1000 population).

Supplementary Table 4: Sensitivity analysis presenting main findings further adjusted for data zone-level average income deprivation and change in income deprivation.

Supplementary Table 5: Sensitivity analysis presenting main findings among individuals who stayed at the same residential data zone during the entire study period.

Supplementary Table 6: Sensitivity analysis presenting main findings after excluding participants with psychiatric inpatient service use in 2001/09 and psychotropic medications in 2009.

Supplementary Table 1: Description of the covariates.[a]

Variable	Description
Sex in 2001	Male, Female
Age in 2001	Derived from the date of birth question
Ethnicity in 2001	
White	White Scottish, Other White British, White Irish, Other White
Non-white	Any Mixed Background, Indian, Pakistani, Bangladeshi, Other South Asian, Caribbean, African, Black Scottish or Other Black, Chinese, Other Ethnic Group
Educational attainment 2001/2011	
No qualification	No professional, vocational or academic qualifications
Level 1	O Grade, Standard Grade, Access 3 Cluster, Intermediate 1 or 2, GCSE, CSE, Senior Certificate or equivalent; GSVQ Foundation or Intermediate, SVQ level 1 or 2, SCOTVEC Module, City and Guilds Craft or equivalent; Other school qualifications not already mentioned (including foreign qualifications)
Level 2	SCE Higher Grade, Higher, Advanced Higher, CSYS, A Level, AS Level, Advanced Senior Certificate or equivalent; GSVQ Advanced, SVQ level 3, ONC, OND, SCOTVEC National Diploma, City and Guilds Advanced Craft or equivalent
Level 3	HNC, HND, SVQ level 4 or equivalent; Other post-school but pre-Higher Education qualifications not already mentioned (including foreign qualifications)
Level 4	Degree, Postgraduate qualifications, Masters, PhD, SVQ level 5 or equivalent; Professional qualifications (for example, teaching, nursing, accountancy); Other Higher Education qualifications not already mentioned (including foreign qualifications)
Social class based on occupation 2001	
I/II	Professional, managerial and technical occupations
IIIN	Skilled non-manual occupations
IIIM	Skilled manual occupations
IV	Partly skilled occupations
V	Unskilled occupations
Other	Non-resident students and people who never worked
Social grade in 2011[b]	
AB	Higher or intermediate managerial, administrative or professional
C1	Supervisory, clerical and junior managerial, administrative and professional
C2	Skilled manual workers
D	Semi-skilled and unskilled manual workers
E	State pensioners, casual and lowest grade workers, unemployed with state benefits only
Employment status 2001/2011	
In employment	Economically active: In employment (part-time, full-time)
Retired	Economically inactive: Retired
Out of labour force	Economically inactive: Student; Looking after home or family; Long-term sick or disabled; Other
Unemployed	Economically active: Unemployed (Seeking work and available to start in 2 weeks or waiting to start a job already obtained)
Marital status in 2001/2011	
Married	Married; In a registered same-sex civil partnership
Single	Never married and never registered a same-sex civil partnership
Separated	Separated, but still legally married; Separated, but still legally in a same-sex civil partnership

Divorced	Divorced; Formerly in a same-sex civil partnership which is now legally dissolved
Widowed	Widowed; Surviving partner from a same-sex civil partnership
Living status in 2001/2011	
Alone	One Person Household
With others	Other Households
Long-term illness in 2001	
Yes	Has a long-term illness, health problem or disability which limits daily activities or work (including problems which are due to old age)
No	Does not a long-term illness, health problem or disability which limits daily activities or work (including problems which are due to old age)

[a] Source: https://sls.lscs.ac.uk/variables

[b] http://www.nrs.co.uk/nrs-print/lifestyle-and-classification-data/social-grade/

Supplementary Table 2: Sample size and percentage of individuals with mental health problems by age and moving status.

	Total sample (n=112 251)		Stayers (n=80 958)		Past movers[a] (n=15 940)		Recent movers[b] (n=15 353)	
	n	% of cases	n	% of cases	n	% of cases	n	% of cases
**Self-reported mental-health**								
16-30 years old	30000[c]	5	15000[c]	5	7000[c]	5	8000[c]	7
31-45 years old	45000[c]	6	34000[c]	5	6000[c]	6	5000[c]	9
46-60 years old	37000[c]	4	32000[c]	4	3000[c]	5	2000[c]	6
**Antidepressant prescriptions**								
16-30 years old	30000[c]	12	15000[c]	12	7000[c]	12	8000[c]	13
31-45 years old	45000[c]	15	34000[c]	14	6000[c]	16	5000[c]	21
46-60 years old	37000[c]	15	32000[c]	15	3000[c]	19	2000[c]	20
**Antipsychotic prescriptions**								
16-30 years old	30000[c]	1	15000[c]	1	7000[c]	1	8000[c]	1
31-45 years old	45000[c]	1	34000[c]	1	6000[c]	1	5000[c]	3
46-60 years old	37000[c]	1	32000[c]	1	3000[c]	1	2000[c]	2

Source: Scottish Longitudinal Study.
Note: Age cohorts relate to age in 2001.
[a] Main residential location changed between 2004/06 and 2007/09.
[b] Main residential location changed between 2007/09 and 2010/12.
[c] Numbers are rounded to avoid risk of disclosure.

Supplementary Table 3: Average crime exposure and change in crime by age and moving status (crime per 1000 population).

	Total sample (n=112 251)		Stayers (n=80 958)		Past movers[a] (n=15 940)		Recent movers[b] (n=15 353)	
	Average crime exposure (SD)	Change in crime exposure (2007/09-2010/12)	Average crime exposure (SD)	Change in crime exposure (2007/09-2010/12)	Average crime exposure (SD)	Change in crime exposure (2004/06-2007/09)	Average crime exposure (SD)	Change in crime exposure (2007/09-2010/12)
16-30 years old	49.2 (53.2)	-8.3 (34.4)	46.8 (57.5)	-6.8 (17.3)	49.6 (47.6)	-10.1 (64.1)	53.3 (49.4)	-10.3 (55.0)
31-45 years old	42.8 (45.5)	-6.6 (22.6)	41.4 (45.6)	-6.1 (15.1)	45.3 (42.6)	-8.2 (50.8)	49.4 (47.1)	-8.7 (47.3)
46-60 years old	41.9 (43.4)	-6.1 (19.3)	41.1 (43.5)	-6.0 (14.4)	45.4 (39.9)	-7.1 (47.8)	48.6 (44.0)	-7.0 (48.4)
All ages	44.2 (47.1)	-6.9 (25.5)	42.3 (47.3)	-6.2 (15.3)	47.3 (44.6)	-9.0 (57.0)	51.3 (47.9)	-9.3 (51.6)

Source: Scottish Longitudinal Study.
Note: Age cohorts relate to age in 2001. SD, Standard deviation.
[a] Main residential location changed between 2004/06 and 2007/09.
[b] Main residential location changed between 2007/09 and 2010/12.

Supplementary Table 4: Sensitivity analysis presenting main findings further adjusted for data zone-level average income deprivation and change in income deprivation.

	Total sample (n=112 251)		Stayers (n=80 958)		Past movers[a] (n=15 940)		Recent movers[b] (n=15 353)	
	Average crime exposure ($\bar{x}$)	Change in crime exposure ($x_{\Delta 2010/12}$)	Average crime exposure ($\bar{x}$)	Change in crime exposure ($x_{\Delta 2010/12}$)	Average crime exposure ($\bar{x}$)	Change in crime exposure ($x_{\Delta 2004/06}$)	Average crime exposure ($\bar{x}$)	Change in crime exposure ($x_{\Delta 2010/12}$)
**Self-reported mental-health**								
16-30 years old	**1.42 (1.08-1.87)**	1.01 (0.99-1.04)	**1.39 (1.00-1.94)**	1.08 (0.98-1.20)	1.20 (0.66-2.18)	1.01 (0.96-1.06)	1.39 (0.91-2.11)	1.00 (0.96-1.04)
31-45 years old	1.01 (0.84-1.23)	1.04 (0.99-1.08)	0.94 (0.74-1.20)	0.99 (0.90-1.09)	0.94 (0.53-1.68)	1.03 (0.96-1.11)	0.99 (0.59-1.66)	*1.06 (0.99-1.13)*
46-60 years old	1.11 (0.87-1.42)	0.98 (0.94-1.03)	1.09 (0.83-1.42)	0.96 (0.87-1.05)	1.11 (0.54-2.31)	1.02 (0.95-1.09)	0.55 (0.21-1.39)	0.99 (0.90-1.10)
All ages	1.12 (0.95-1.33)	1.02 (0.99-1.04)	1.07 (0.90-1.27)	1.00 (0.93-1.08)	1.08 (0.75-1.55)	1.01 (0.98-1.04)	1.07 (0.74-1.56)	1.02 (0.99-1.05)
**Antidepressant prescriptions**								
16-30 years old	1.03 (0.86-1.24)	1.01 (0.99-1.03)	1.07 (0.86-1.34)	**1.07 (1.02-1.12)**	0.90 (0.60-1.34)	1.01 (0.97-1.05)	0.91 (0.69-1.20)	0.99 (0.96-1.03)
31-45 years old	0.98 (0.87-1.10)	1.00 (0.96-1.03)	0.90 (0.77-1.06)	0.99 (0.92-1.05)	0.87 (0.65-1.17)	1.02 (0.98-1.05)	1.29 (0.86-1.93)	1.00 (0.96-1.03)
46-60 years old	1.05 (0.94-1.18)	0.98 (0.95-1.02)	1.06 (0.95-1.19)	0.97 (0.92-1.01)	1.00 (0.58-1.72)	0.96 (0.89-1.03)	**0.55 (0.34-0.89)**	1.00 (0.93-1.08)
All ages	1.02 (0.93-1.12)	1.00 (0.98-1.02)	1.00 (0.91-1.10)	0.99 (0.96-1.02)	0.92 (0.71-1.20)	1.00 (0.98-1.03)	0.94 (0.75-1.18)	0.99 (0.96-1.03)
**Antipsychotic prescriptions**								
16-30 years old	1.27 (0.78-2.05)	1.01 (0.92-1.11)	1.39 (0.67-2.87)	1.24 (0.89-1.74)	1.46 (0.47-4.54)	1.04 (0.98-1.10)	0.87 (0.37-2.07)	0.94 (0.88-1.02)
31-45 years old	1.15 (0.72-1.83)	*1.10 (1.00-1.21)*	1.02 (0.63-1.68)	1.03 (0.88-1.22)	0.75 (0.22-2.62)	1.04 (0.93-1.16)	1.95 (0.71-5.36)	**1.14 (1.03-1.26)**
46-60 years old	1.53 (1.04-2.26)	1.05 (0.96-1.15)	**1.80 (1.13-2.87)**	1.14 (0.95-1.37)	0.46 (0.10-2.10)	0.96 (0.83-1.10)	0.63 (0.16-2.45)	1.04 (0.89-1.22)
All ages	**1.27 (1.01-1.59)**	**1.05 (1.00-1.10)**	**1.37 (1.03-1.82)**	1.10 (0.98-1.24)	0.93 (0.47-1.86)	1.02 (0.95-1.09)	1.21 (0.64-2.28)	1.05 (0.98-1.12)

Source: Scottish Longitudinal Study.

Note: Age cohorts relate to age in 2001. Bold text indicates significant associations ($p < 0.05$), italic trend-wise ($p < 0.1$). Average crime exposure is log10-transformed, change in crime exposure is standardized. Models were fitted with logistic regression applying cluster robust estimation at local authority level; estimates are expressed in OR with 95% CI. All models included average and change variables at the same time, and were adjusted for sex, age (and age-squared in the non-stratified total sample), 2001 baseline covariates (ethnicity; education; social class; employment; marital status; living status; long-term illness), psychiatric inpatient service use in 2001/03, 2001 – 2011 change indicators (gained higher level of education; separated, divorced or widowed; started to live alone; became unemployed or left labour force) and social grade in 2011.

[a] Main residential location changed between 2004/06 and 2007/09.

[b] Main residential location changed between 2007/09 and 2010/12.

Supplementary Table 5: Sensitivity analysis presenting main findings among individuals who stayed at the same residential data zone during the entire study period.

	Stayers (n=71 000)[a]	
	Average crime exposure ($\bar{x}$)	Change in crime exposure ($x_{\Delta 2010/12}$)
**Self-reported mental-health**		
16-30 years old	**1.72 (1.29-2.30)**	*1.10 (0.99-1.22)*
31-45 years old	**1.29 (1.03-1.62)**	1.01 (0.91-1.11)
46-60 years old	**1.32 (1.07-1.63)**	0.95 (0.86-1.06)
All ages	**1.38 (1.20-1.59)**	1.01 (0.93-1.09)
**Antidepressant prescriptions**		
16-30 years old	**1.39 (1.15-1.66)**	*1.05 (0.99-1.12)*
31-45 years old	**1.18 (1.05-1.34)**	1.00 (0.93-1.08)
46-60 years old	**1.26 (1.15-1.38)**	0.97 (0.93-1.02)
All ages	**1.26 (1.17-1.35)**	1.00 (0.96-1.03)
**Antipsychotic prescriptions**		
16-30 years old	1.17 (0.71-1.92)	1.12 (0.75-1.66)
31-45 years old	1.13 (0.74-1.73)	1.07 (0.89-1.28)
46-60 years old	**1.45 (1.05-2.00)**	1.14 (0.96-1.34)
All ages	**1.28 (1.02-1.60)**	1.10 (0.98-1.25)

Source: Scottish Longitudinal Study.
Note: Age cohorts relate to age in 2001. Bold text indicates significant associations ($p<0.05$), italic trend-wise ($p<0.1$). Average crime exposure is log10-transformed, change in crime exposure is standardized. Models were fitted with logistic regression applying cluster robust estimation at local authority level; estimates are expressed in OR with 95% CI. All models included average and change variables at the same time, and were adjusted for sex, age (and age-squared in the non-stratified total sample), 2001 baseline covariates (ethnicity; education; social class; employment; marital status; living status; long-term illness), psychiatric inpatient service use in 2001/03, 2001 – 2011 change indicators (gained higher level of education; separated, divorced or widowed; started to live alone; became unemployed or left labour force) and social grade in 2011.
[a] Number is rounded to avoid risk of disclosure.

Supplementary Table 6: Sensitivity analysis presenting main findings after excluding participants with psychiatric inpatient service use in 2001/09 and psychotropic medications in 2009

	Total sample (n=93 000)[a]		Stayers		Past movers[b]		Recent movers[c]	
	Average crime exposure ($\bar{x}$)	Change in crime exposure ($x_{\Delta 2010/12}$)	Average crime exposure ($\bar{x}$)	Change in crime exposure ($x_{\Delta 2010/12}$)	Average crime exposure ($\bar{x}$)	Change in crime exposure ($x_{\Delta 2004/06}$)	Average crime exposure ($\bar{x}$)	Change in crime exposure ($x_{\Delta 2010/12}$)
**Self-reported mental-health**								
16-30 years old	**1.68 (1.28-2.21)**	1.03 (0.99-1.07)	**1.76 (1.16-2.68)**	1.08 (0.92-1.26)	1.48 (0.84-2.62)	*1.05 (1.00-1.11)*	1.54 (0.83-2.88)	1.02 (0.96-1.09)
31-45 years old	**1.84 (1.40-2.43)**	1.05 (0.98-1.12)	**1.77 (1.27-2.46)**	1.02 (0.83-1.25)	1.55 (0.79-3.05)	1.00 (0.94-1.06)	*1.87 (0.93-3.74)*	*1.08 (1.00-1.17)*
46-60 years old	**1.50 (1.05-2.14)**	0.99 (0.91-1.07)	1.34 (0.93-1.93)	0.90 (0.75-1.07)	1.25 (0.63-2.49)	1.06 (0.98-1.14)	2.47 (0.58-10.54)	1.01 (0.82-1.25)
All ages	**1.71 (1.44-2.04)**	1.03 (0.99-1.06)	**1.66 (1.33-2.07)**	1.00 (0.88-1.14)	**1.59 (1.09-2.30)**	**1.03 (1.00-1.06)**	**1.73 (1.08-2.78)**	*1.04 (0.99-1.08)*
**Antidepressant prescriptions**								
16-30 years old	**1.20 (1.02-1.41)**	1.03 (0.99-1.06)	**1.27 (1.01-1.60)**	**1.12 (1.04-1.21)**	1.26 (0.92-1.73)	1.01 (0.97-1.05)	1.00 (0.75-1.34)	1.02 (0.98-1.06)
31-45 years old	**1.22 (1.07-1.39)**	0.99 (0.95-1.03)	*1.16 (0.99-1.35)*	0.99 (0.87-1.13)	1.29 (0.91-1.83)	0.97 (0.94-1.01)	**1.53 (1.04-2.25)**	1.00 (0.96-1.04)
46-60 years old	*1.19 (0.99-1.41)*	0.98 (0.91-1.07)	**1.26 (1.04-1.54)**	1.01 (0.91-1.11)	0.85 (0.51-1.42)	**0.92 (0.85-1.00)**	0.64 (0.31-1.34)	0.99 (0.87-1.12)
All ages	**1.22 (1.12-1.34)**	1.01 (0.98-1.03)	**1.24 (1.14-1.35)**	1.03 (0.96-1.10)	**1.21 (1.00-1.46)**	*0.98 (0.96-1.00)*	1.09 (0.85-1.39)	1.01 (0.97-1.04)
**Antipsychotic prescriptions**								
16-30 years old	**3.26 (1.03-10.27)**	0.97 (0.85-1.11)	**4.55 (1.31-15.88)**	**1.59 (1.07-2.37)**	[d]	[d]	[d]	[d]
31-45 years old	**2.16 (1.04-4.47)**	1.03 (0.86-1.23)	1.58 (0.60-4.16)	0.94 (0.77-1.15)	[d]	[d]	[d]	[d]
46-60 years old	1.34 (0.29-6.18)	0.81 (0.60-1.07)	1.15 (0.20-6.65)	0.94 (0.37-2.41)	[d]	[d]	[d]	[d]
All ages	**2.33 (1.22-4.44)**	0.96 (0.91-1.02)	2.07 (0.84-5.07)	1.05 (0.72-1.53)	[d]	[d]	[d]	[d]

Source: Scottish Longitudinal Study.

Note: Age cohorts relate to age in 2001. Bold text indicates significant associations ($p < 0.05$), italic trend-wise ($p < 0.1$). Average crime exposure is log10-transformed, change in crime exposure is standardized. Models were fitted with Poisson regression applying cluster robust estimation at local authority level; estimates are expressed in IRR with 95% CI. All models included average and change variables at the same time, and were adjusted for sex, age (and age-squared in the non-stratified total sample), 2001 baseline covariates (ethnicity; education; social class; employment; marital status; living status; long-term illness), psychiatric inpatient service use in 2001/03, 2001 – 2011 change indicators (gained higher level of education; separated, divorced or widowed; started to live alone; became unemployed or left labour force) and social grade in 2011.

[a] Number is rounded to avoid risk of disclosure.

[b] Main residential location changed between 2004/06 and 2007/09.

[c] Main residential location changed between 2007/09 and 2010/12.

[d] Could not be estimated because of the very low number of cases.
